# Clinical Veterinarian Perspectives on Euthanasia for Pets

**DOI:** 10.3390/ani16213349

**Published:** 2026-10-23

**Authors:** Emine Merve Danış, Aşkın Yaşar

**Affiliations:** Faculty of Veterinary Medicine, Selçuk University, Konya 42130, Türkiye; ayasar@selcuk.edu.tr

**Keywords:** animal welfare, euthanasia, ethical decision-making, pet animals, clinical veterinarian, veterinary ethics

## Abstract

The euthanasia of pets is a frequent discussion topic in veterinary practice. This study aimed to determine the levels of agreement with euthanasia decisions for pet animals by clinical veterinarians in different clinical scenarios; evaluate the differences in agreement levels according to species (including cats, dogs, and small mammals); and investigate the correlation between these decisions and demographic variables. The study sample consisted of 374 veterinarians actively practicing in clinical veterinary medicine in Türkiye. Data were collected via an online survey administered to the participants. The attitudes of participants toward euthanasia varied according to the animal species and case characteristics. The agreement with euthanasia decisions significantly varied in certain cases based on characteristics, such as the participants’ professional experience, pet ownership status, and prior experience with performing euthanasia. Thus, the euthanasia decision is a multidimensional process in which clinical, ethical, and individual factors are simultaneously evaluated. A detailed examination of the ethical, professional, and individual rationales underlying euthanasia decisions through qualitative studies is warranted based on the case scenarios used in this research, which can benefit pets, veterinarians, and animal owners.

## Simple Summary

The euthanasia of companion animals is a major issue in veterinary science. This study examined veterinarians’ agreement with euthanasia in companion animals across various clinical scenarios. A total of 374 veterinarians actively practicing clinical veterinary medicine in Türkiye participated in the study, and data were collected through an online questionnaire. The willingness of participants to perform euthanasia varied depending on the animal species (cat, dog, and small mammal) and the specific characteristics of the case. In some cases, this willingness varied based on participant characteristics, such as professional experience, pet ownership status, and prior experience performing euthanasia. Thus, the decision to perform euthanasia involves the simultaneous consideration of clinical, ethical, and individual factors. Future qualitative studies must focus on the case scenarios used in this research to examine the ethical, professional, and personal rationales underlying euthanasia decisions, which can benefit pet animals, veterinarians, and pet owners.

## 1. Introduction

According to Yeates [1], euthanasia is a common and central component of most areas of veterinary practice. Veterinary euthanasia refers to “ending an animal’s life in a manner that minimizes or eliminates pain and distress” [2,3]. The justification for killing animals is a major ethical issue. Euthanasia may appear justifiable in various contexts, such as slaughter, self-defense, culling, or killing for research and testing [3]. However, some researchers argue that the term “euthanasia” should be restricted to practices that serve the animal’s best interests. Quain [4] defines ethically appropriate euthanasia as a procedure carried out in the animal’s interest, where the primary motivation is not convenience, economic reasons, or grounds deemed ethically unacceptable by the veterinary team. Tom Regan, meanwhile, posits that ethically appropriate euthanasia must be performed using the least painful method possible, be based on a valid belief that it is in the animal’s best interest, and be driven by the practitioner’s commitment to the animal’s interests and welfare [5].

Several factors, such as socioeconomic, emotional, and pathological factors, influence the decision on euthanasia [6]. Pet owners may request euthanasia for reasons such as no longer wanting the animal, an inability to continue its care, behavioral problems, or dangerous aggression [7].

Ethical values and decision-making frameworks can support veterinarians in their decisions on the euthanasia process and facilitate animal owners in coping with ethical dilemmas [8]. The decision to euthanize an animal requires addressing the situation from medical, legal, ethical, and moral perspectives [6], considering the quality of life of animals, the wishes of the owner, and the professional responsibilities of veterinarians [9]. These requirements may vary depending on the field of veterinary medicine. For example, veterinarians consider the quality of life of pets and the emotional bond of owners. In contrast, veterinarians generally balance the welfare of farm animals with the economic interests of farmers [6]. However, the frequency and variety of euthanasia cases may vary depending on both the legal framework of a country and the working environment of veterinarians [3].

Legal regulations regarding animal euthanasia in Türkiye are set forth in Law No. 5996 on Veterinary Services, Plant Health, Food and Feed [10], Law No. 5199 on Animal Protection [11], and the Implementing Regulation on Animal Protection [12]. Under Article 9 of Law No. 5996, euthanasia is generally prohibited. However, a veterinarian may decide to perform euthanasia in cases such as illnesses that cause animal pain and suffering or for which there is no possibility of recovery; the control of certain infectious diseases; and uncontrollable behaviors that pose a threat to human or animal health. The euthanasia procedure is performed by a veterinarian or under the supervision of a veterinarian [10]. Furthermore, Article 13 of Law No. 5199 contains specific provisions regarding dogs that meet certain criteria and are admitted to animal shelters [11]. The relevant implementing regulation sets forth the conditions regarding the methods to be used in transactions covered by this article and prohibits certain methods [12]. Economic reasons, however, are not explicitly stated as grounds for euthanasia in the relevant legislative texts. In Türkiye, animals have played various roles in social, economic, and cultural life, while traditional beliefs and cultural practices have also shaped human–animal relationships [13]. However, although legal regulations concerning animal euthanasia exist in Türkiye, research directly examining the general public’s views on this issue remains limited [14,15].

According to the FEDIAF annual report [16], Türkiye has 19,192,000 pets (4,887,000 cats, 1,453,000 dogs, 11,840,000 birds, and 847,000 small mammals), excluding aquarium animals. The increase in the number of pets worldwide, including Türkiye [16,17], affects the healthcare services and care processes provided by veterinarians for pets [18].

Studies examining views on euthanasia in Türkiye have addressed the attitudes of veterinary students toward the practice [14] and there is also qualitative research investigating the decision-making processes of veterinarians regarding euthanasia [15]. However, participant groups and research methodologies vary across these previous studies. The scarcity of research in Türkiye that comparatively examines how clinical veterinarians evaluate euthanasia across different clinical case scenarios—specifically involving cats, dogs, and small mammals—highlights a gap in the literature. Furthermore, the prevalence of cats, dogs, and small mammals in Türkiye’s pet population makes it meaningful to consider these species together in veterinarians’ euthanasia assessments. This study aimed to determine the attitudes of clinical veterinarians in Türkiye toward euthanasia of pets in various scenarios, evaluate these attitudes toward different animal species, and reveal the relationship between these decisions and demographic variables.

## 2. Materials and Methods

Development of the questionnaire

A questionnaire using brief case scenarios (vignette scenarios) was designed to assess the agreement with euthanasia decisions by veterinarians for pet animals in Türkiye. The survey questions were developed by the authors based on literature reviews [3,6,19,20,21,22,23,24]. Vignette scenarios were prepared to reflect different euthanasia situations that veterinarians may encounter, considering medical, financial (of the owner), and ethical factors in which euthanasia may be considered in veterinary medicine. Participants were instructed to evaluate the scenarios as veterinarians in each scenario.

Expert opinions were obtained from eight faculty members working in the fields of veterinary clinical sciences (surgery, internal medicine, and obstetrics) to evaluate the scope, content, and appropriateness of the wording of the developed questionnaire. The questionnaire form was revised based on the feedback. A pilot study was conducted with 25 veterinarians to evaluate the clarity, applicability, and completion time of the questions. The questionnaire form was finalized based on the feedback from the pilot study. The veterinarians participating in the pilot study were excluded from the main study.

The questionnaire used in this study comprised two parts. The first part comprised 12 items to determine sociodemographic characteristics. Meanwhile, the second part comprised 14 vignette scenarios to determine the decisions of veterinarians on euthanasia requests for the relevant animal. Participants were instructed to provide separate assessments for cats, dogs, and small mammals. Separate response fields were created for the relevant animal species in each scenario.

A five-point Likert scale was used to evaluate the vignette scenarios. Based on the scenarios, veterinarians’ agreement with performing euthanasia on the specific animal described in each scenario was assessed using a five-point Likert scale with the following response options and corresponding scores: “strongly disagree” (1), “disagree” (2), “undecided” (3), “agree” (4), and “strongly agree” (5).

To facilitate the presentation of the findings, the vignette scenarios were coded as “Case 1, …, Case 14.” Questions 13–26 were designated as “Case 1, …, Case 14,” respectively. The code and scenario content for each case are presented below:

“*Case 1: I would agree to an owner’s request for euthanasia for an animal that continues to exhibit aggressive behavior and has previously injured people despite training and behavioral therapy.*

*Case 2: I would agree to the request of an animal shelter that houses animals far beyond its capacity and seeks euthanasia for the animals due to limited staff and housing space.*

*Case 3: I would agree to an owner’s request for euthanasia for an animal a few months old that has a hereditary disease preventing it from eating.*

*Case 4: I would agree to the animal owner’s request for euthanasia instead of ovariohysterectomy for a breeding animal diagnosed with a uterine tumor and recommended to undergo ovariohysterectomy, because the animal would no longer have breeding value.*

*Case 5: I would agree to the request for euthanasia from an animal owner whose animal has no health condition that threatens its life but who requests euthanasia and states that, if the request is refused, they will end the animal’s life by their own means.*

*Case 6: I would agree to the request of an animal owner who seeks euthanasia to relieve the suffering of an animal with a treatable disease that is, however, too costly for the owner to manage.*

*Case 7: I would agree to the request for euthanasia from an animal owner whose animal, due to its advanced age, is unable to adapt to the owner’s living conditions and whom the owner does not wish to surrender to an animal shelter.*

*Case 8: I would agree to the requests from members of the public to euthanize the offspring of stray or unowned animals to prevent them from growing and reproducing uncontrollably.*

*Case 9: I would agree to the request for euthanasia from an animal owner who does not wish to continue treatment for an animal with an incurable chronic disease, despite the possibility of improving the animal’s quality of life with appropriate analgesic treatment.*

*Case 10: I would agree to the request for euthanasia from an animal owner who states that they cannot afford the treatment costs for an animal that has sustained severe injuries in a traffic accident and is at risk of death if left untreated, in order to alleviate the animal’s suffering.*

*Case 11: I would agree to the request of an animal owner who seeks euthanasia to alleviate the suffering of their animal, which is battling various health issues and whose condition has deteriorated despite prolonged treatment.*

*Case 12: Even if I do not consider euthanasia medically appropriate, I would agree to the request for euthanasia in situations where refusing the request could damage my relationship with the animal owner.*

*Case 13: I would agree to the request for euthanasia from an animal owner who, because the treatment of their animal’s treatable disease is costly and time-consuming, prefers to purchase a new animal rather than spend money on treatment and therefore requests euthanasia for the existing animal.*

*Case 14: I would agree to the request of an animal owner seeking euthanasia for an animal suffering from a zoonotic disease.*”

Study population and sample

The study population comprised clinical veterinarians actively working in animal hospitals, polyclinics, and veterinary practices in Türkiye as of 2025. To determine the total number of clinical veterinarians constituting the study population, data on the number of animal hospitals, polyclinics, and veterinary practices in Türkiye, published by the Turkish Ministry of Agriculture and Forestry (MAF) [25] on 6 January 2025, were used. Data from the MAF and the requirement for the employment of at least five veterinarians in animal hospitals, at least four in polyclinics, and at least one in veterinary practices [26,27] were evaluated. The study population was determined to be 10,186. The required sample size was determined using the formula proposed by Krejcie and Morgan [28], with a 95% confidence level (χ^2^ = 3.841), a population proportion of 0.50, and a margin of error of 0.05. Based on the study population of 10.186 clinical veterinarians, the minimum required sample size was calculated as 371. The following participants were included in the study: practicing veterinarians, veterinarians who voluntarily agreed to participate in the research, and veterinarians who completed the questionnaire.

Data were collected via an online survey prepared using the Google Forms platform. The survey link was shared via social media platforms, veterinary professional chambers, and professional communication networks. Additionally, the survey link was electronically transmitted to veterinarians whose contact information was available. The survey link was redistributed at three-month intervals throughout the data collection process along with reminder posts. Data were collected from 374 veterinarians between 23 June 2025, and 20 April 2026. All questionnaire items were set as mandatory in Google Forms, preventing respondents from proceeding without answering each question. Consequently, no missing responses were present in the dataset included in the analyses.

Statistical analyses

All statistical analyses were performed using IBM SPSS Statistics 29.0 (IBM Corp., Armonk, NY, USA). Normality was assessed using the Shapiro–Wilk test and graphical methods. The distributions of the study variables deviated significantly from normality (*p* < 0.001); therefore, non-parametric tests were used. Descriptive statistics for Likert-scale items are presented as medians and interquartile ranges (Q1–Q3). Categorical variables are presented as frequencies and percentages. Non-parametric tests were used for group comparisons because the data did not follow a normal distribution.

Differences between two independent groups were assessed using the Mann–Whitney U test, whereas differences among more than two independent groups were assessed using the Kruskal–Wallis H test. When the Kruskal–Wallis H test indicated a statistically significant difference, pairwise comparisons were performed using Dunn’s post hoc test. Comparisons among cats, dogs, and small mammals, which were evaluated by the same respondents, were performed using the Friedman test. When the Friedman test indicated a statistically significant difference, pairwise comparisons were conducted using the Durbin–Conover procedure. Differences were considered statistically significant at *p* < 0.05.

The association between years of clinical experience and previous euthanasia experience was examined using Pearson’s chi-square test of independence.

## 3. Results

Of the 374 veterinarians, 212 (56.7%) were males, and 162 (43.3%) were females. Participants in the age group of 22–33 years accounted for the largest proportion (45.2%; *n* = 169), whereas those in the age group of ≥56 years accounted for the smallest proportion (3.5%; *n* = 13). The work environment of the participants was as follows: veterinary practices, 73.0% (*n* = 273); polyclinics, 12.8% (*n* = 48); and animal hospitals, 14.2% (*n* = 53). Participants with 5–10 years of experience accounted for the largest proportion (38.5%; *n* = 144), whereas those with ≥22 years of experience accounted for the smallest proportion (8.3%; *n* = 31). Veterinarians who are also pet owners constituted 70.6% of the participants (*n* = 264) (Table 1).

Analysis of views on euthanasia of pet animals revealed that 54.0% (*n* = 202) of veterinarians considered it a necessary but difficult procedure, while 11.8% (*n* = 44) considered it necessary for animal welfare. None of the participants viewed euthanasia as a routine procedure. The proportions of participants who previously performed euthanasia on a pet and their own pet were 69.0% (*n* = 258) and 14.6% (*n* = 29), respectively (Table 1).

When veterinarians’ agreement with euthanasia requests was evaluated across animal species (cats, dogs, and small mammals), statistically significant differences between species were found in Cases 1–2, 4–9, and 12–13 (*p* < 0.05). In contrast, no statistically significant differences were observed in Cases 3, 10–11, and 14 (*p* > 0.05), indicating similar levels of agreement across species in these cases (Table 2). Pairwise post hoc comparisons revealed that agreement scores were significantly higher for dogs in Cases 1–2 and 8, for small mammals in Cases 4–6, 9, and 12–13, and for both dogs and small mammals in Case 7, compared with the other species (*p* < 0.05; Table 2).

### Evaluation Based on Sociodemographic Variables

Euthanasia decisions did not significantly vary according to gender across all cases (cases 1–14) and evaluated species (cats, dogs, and small mammals) (*p* > 0.05; Supplementary Table S1). Similarly, euthanasia decisions did not significantly vary based on the work environment of veterinarians across all presented cases (cases 1–14) and evaluated species (cats, dogs, and small mammals) (*p* > 0.05; Supplementary Table S2).

Euthanasia decisions significantly varied based on the pet ownership status of participants in 13 cases (cases 1–10 and 12–14) involving cats, dogs, and small mammals (*p* < 0.05; Table 3) but were not significant in one case (case 11) (*p* > 0.05; Table 3). In all cases with significant differences, veterinarians who did not own pets had higher scores for agreement with euthanasia requests than those who owned pets (Table 3).

Agreement with euthanasia decisions significantly varied based on the duration of professional experience for cases 1–2, case 3 (cats only), cases 4–8, case 9 (small mammals only), case 10, and cases 12–13 (*p* < 0.05; Table 4). For case 3 (dogs and small mammals), case 9 (cats and dogs), case 11, and case 14, the levels of agreement were similar (*p* > 0.05; Table 4). A detailed distribution of pairwise comparison (post-hoc) results is presented in Table 4.

Agreement with euthanasia decisions significantly varied based on prior experience with euthanasia performed across all species in cases 3, 11, and 14 (*p* < 0.05; Table 5). In case 8, the scores for cats and small mammals, but not for dogs, significantly varied. The differences were also not significant in the other examined cases (cases 1–2, 4–7, 9–10, and 12–13) (*p* > 0.05). Only comparisons showing statistically significant differences are presented in Table 5.

The relationship between years of clinical experience and previous euthanasia experience was examined. No statistically significant association was found between clinical experience duration and previous euthanasia experience (χ^2^ = 1.068, df = 3, *p* = 0.785).

## 4. Discussion

This study evaluated veterinarians’ agreement with euthanasia across different cases involving cats, dogs, and small mammals. Agreement varied according to the case and certain demographic variables (Table 2, Table 3, Table 4 and Table 5, Supplementary Tables S1 and S2).

Rollin [29] revealed that the aim of veterinary medicine is to restore and maintain the health of animals. Additionally, putting an end to pain or suffering that completely destroys the quality of life of animals is conceptually a part of the veterinarian’s duty. According to de Santis Bastos and Cohen [30], although several veterinarians consider euthanasia clinically and ethically necessary and appropriate, they experience stress and suffer serious psychological harm from the process because they feel like “executioners.” In this study, participating veterinarians most frequently (54%) characterized euthanasia as a “necessary but difficult-to-perform procedure” (Table 1). This finding is consistent with the views of Rollin [29] and the findings of Santis Bastos and Cohen [30]. Thus, the ethical responsibility and emotional burden accompanying the decision to end the life of animals are crucial for the assessments of veterinarians. However, none of the participants considered euthanasia “a routine procedure” (Table 1), which indicates that euthanasia is not trivialized by veterinarians and that they professionally evaluate the decision. Furthermore, a total of 17.6% of participants stated that “a procedure that a veterinarian should not perform” (Table 1). This finding indicates differing views on the role of euthanasia in veterinary practice. However, the reasons underlying these views were not directly investigated in the present study. Although the circumstances under which euthanasia may be performed in Türkiye are defined by legal regulations, the absence of an explicit provision regarding veterinarians’ refusal of euthanasia requests highlights the importance of addressing this issue from legal, professional, and ethical perspectives.

One study [31] reported that behavioral problems were the reason for euthanasia in 13% of dogs and one cat. Behavioral disorders are among the most prominent reasons for euthanasia in dogs [32]. In particular, aggression toward humans is the most common reason for behavioral euthanasia [33]. In this study, the levels of agreement with euthanasia decisions of veterinarians were significantly high for dogs in cases involving aggressive behavior and the uncontrolled reproduction of stray/street animals (case 1 and case 8) (*p* < 0.05) (Table 2), which is consistent with the findings reported in the literature. However, the reasons influencing veterinarians’ agreement with these decisions were not directly investigated in the present study. The role of factors such as public health, human safety, and the risk of zoonotic disease transmission in veterinarians’ agreement with euthanasia decisions could be examined in future research.

Bradley and Rajendran [34] reported that 50% of animals entering shelters each year are euthanized and that 10–25% of these animals are euthanized specifically due to overcrowding in the shelters. Recently, animal populations in U.S. shelters have rapidly increased, contributing to enhanced euthanasia rates. Both cats and dogs are at risk of euthanasia. However, dogs are at an increased risk of euthanasia [35]. Overcrowding, limited capacity, and a lack of resources in shelters lead to animals being housed in crowded, noisy, and stressful conditions [36], adversely affecting their welfare [36,37]. Euthanasia is used to combat the overpopulation of stray dogs in regions with insufficient shelter capacity and resources, even in high-income countries [38]. In the case involving insufficient shelter capacity (case 2), veterinarians’ agreement scores for euthanasia decisions were significantly higher for dogs (*p* < 0.05; Table 2). This can be attributed to overcrowding in shelters, which may compromise animal welfare by increasing spatial stress and the risk of injury among dogs, potentially influencing the euthanasia assessments of veterinarians. Furthermore, dogs are associated with increased challenges related to population control and shelter capacity. Thus, veterinarians may have higher levels of agreement with euthanasia decisions for dogs than for cats and small mammals.

In the long-standing relationship between humans and animals, human perception of and interaction with various animal species vary [39]. Veterinary ethics is associated with various challenges due to the diverse socio-moral positions of animals in modern society and the complex nature of the practice [9]. The perceptions of the similarity between humans and other animal species have a significant impact on animal welfare. Perceived similarity is directly proportional to the level of empathy [40], influencing the care provided to the animal [39]. The views on the moral status of even companion/pet animals, which are generally considered family members, vary in clinical practice. Veterinarians may consider hamsters to be of lower moral importance than dogs or cats (or may assume that animal owners hold such a view). Additionally, veterinarians and animal owners may differentially value animals. Furthermore, these views may lead to confusion or disagreement [41]. The decision to euthanize species, such as small mammals and reptiles, can be influenced by factors beyond clinical assessment, such as the experience of veterinarians with the species, the circumstances of the animal and its owner, and the available technical and financial resources [42]. Hartnack et al. [21] reported that agreement with euthanasia for rabbit euthanasia is lower than that for dog euthanasia. Evidence for rabbits being considered morally less important than dogs is lacking. In this study, veterinarians’ agreement scores for euthanasia decisions were significantly high for small mammals in the following cases: loss of the breeding value of animals (case 4), concern that the owner would intervene on their own if the veterinarian refused (case 5), high treatment costs (case 6), concern about damaging the relationship with the owner (case 12), and the idea of acquiring a new animal instead of undergoing costly and lengthy treatment processes (case 13) (*p* < 0.05; Table 2). This finding is consistent with that of previous studies on the moral status of animals and speciesism but is inconsistent with that of Hartnack et al. [21]. These differences observed in relation to small mammals may be discussed in terms of the economic and emotional values attributed to different animal species. However, as these values and speciesism were not directly assessed in the present study, it cannot be conclusively determined whether the observed differences are associated with these factors.

In addition to ending the suffering of animals, euthanasia is a professional decision aimed at ensuring a humane death based on a combined assessment of animal welfare, ethical principles, clinical evaluation, and the circumstances of the animal owner [3]. Kabboush et al. [24], Sugita and Irimajiri [22], and Hartnack et al. [21] reported that female veterinarians exhibit negative attitudes toward euthanasia. However, Dürr et al. [43] revealed no significant differences in attitudes toward euthanasia between male and female veterinarians, except within a narrow age range. The findings of this study were consistent with those of Dürr et al. [43] but not with those of Kabboush et al. [24], Sugita and Irimajiri [22], and Hartnack et al. [21]. In particular, agreement with euthanasia decisions was not significantly different based on gender in any of the evaluated cases (cases 1–14) (Supplementary Table S1). This can be interpreted as limiting the influence of gender-based differences in individual attitudes on the decision. The decision to euthanize is based on professional criteria, such as clinical assessment, animal welfare, and professional responsibility.

Practice and clinical management, economic factors, and the professional autonomy of veterinarians can influence daily working life [44,45,46]. The implementation of standard treatment protocols can limit the autonomy of veterinarians in clinical decision-making processes. Similarly, corporate business models can exert pressure for generating revenue by encouraging veterinarians to focus on the commercial aspects of the profession [46]. Kipperman et al. [23] revealed that veterinarians most frequently used an intuitive approach based on their personal values to resolve ethical dilemmas. Euthanasia decisions were not significantly different based on the work environment across all presented cases (cases 1–14) and all evaluated species (*p* > 0.05; Supplementary Table S2). This finding is consistent with that of Gyles [44], Knights and Clarke [45], Nicol [46], and Kipperman et al. [23]. However, it may be beneficial to examine the potential effects of institutional policies and working conditions on euthanasia decisions in future research.

Animals, especially pets, have a special place and role within the family environment of modern societies. Pets are generally considered a part of the family [47]. The experience of pet ownership is not only a demographic characteristic but also an indicator of a meaningful relationship that fosters a general ethic of care [48]. This relationship encourages helping behaviors by increasing the sensitivity of individuals to the needs of animals and the suffering they experience. Özen et al. [49] reported that the pet ownership status of veterinarians does not alter their empathy levels. In this study, euthanasia decisions significantly varied based on pet ownership status for all species (cats, dogs, and small mammals) (cases 1–10 and 12–14). In particular, veterinarians who did not own pets had higher euthanasia inclination scores than those who owned pets (Table 3). This can be partially attributed to the assessments reported by Colombo et al. [47] and Lu et al. [48] but is not completely consistent with the findings of Özen et al. [49]. The emotional bonds that veterinarians form with their pets may be related to their assessments regarding euthanasia. However, since empathy and emotional bonds were not directly assessed in this study, the observed differences cannot be attributed to these factors.

Previous studies have reported that the level of agreement with euthanasia decisions in various situations varies according to professional experience. Dürr et al. [43] reported that experienced veterinarians were inclined to agree with euthanasia decisions. Conversely, Hartnack et al. [21] and Kabboush et al. [24] emphasized that less experienced veterinarians are likely to oppose euthanasia, including convenience euthanasia. Kabboush et al. [24] reported that experienced physicians can develop effective coping strategies for the emotional burden associated with performing euthanasia. Ouedraogo et al. [50] revealed that experienced physicians experience increased compassion satisfaction and decreased secondary traumatic stress. A study in Türkiye [51] reported that veterinarians with less than five years of active clinical experience had the highest mean ethical sensitivity score, while those with 16–20 years of experience had the lowest mean ethical sensitivity score. In this study, agreement with euthanasia decisions significantly varied based on professional experience in some cases [cases 1–2, case 3 (feline species only), cases 4–8, case 9 (small mammal species only), and cases 10 and 12–13] (*p* < 0.05) but were not significantly different in other cases [case 3 (canine and small mammal species), case 11, and case 14] (*p* > 0.05) (Table 4). Veterinarians in the early stages of their careers (<5 years) exhibited low levels of agreement with euthanasia decisions, which is consistent with the findings of Hartnack et al. [21] and Kabboush et al. [24]. This level of agreement peaked among veterinarians with moderate professional experience (5–10 and 11–21 years). Consistent with the findings of Dürr et al. [43], agreement with euthanasia decisions was low among the most experienced veterinarians (≥22 years) (Table 4). The findings indicate that agreement with euthanasia decisions differed across professional experience groups in some cases and animal species. The lower level of agreement observed among veterinarians in the early stages of their careers may be considered alongside the findings of Danış [51], who reported higher mean ethical sensitivity scores among veterinarians with less than five years of active clinical experience. However, the lower agreement observed among less experienced veterinarians cannot be attributed to professional idealism or heightened ethical sensitivity, as these factors were not directly assessed in the present study. Similarly, the higher agreement among veterinarians with moderate clinical experience and the lower agreement among highly experienced veterinarians should not be interpreted as evidence of changes in attitudes over the course of a veterinary career. Given the cross-sectional design of the present study, these differences may also reflect variations in educational background, cultural values, or other characteristics across the groups.

Yeates and Main [52] revealed that a large proportion of participants did not perform euthanasia for various reasons, such as the animal being young, healthy, or not in a state of suffering. Pugliese et al. [53] reported that congenital diseases are among the primary causes of euthanasia-related deaths. In this study, euthanasia assessments by veterinarians significantly varied according to their prior experience in performing euthanasia on pets in certain cases [case 3, case 8 (in cat and small mammal species), case 11, and case 14] (*p* < 0.05; Table 5). Consistent with the findings of Yeates and Main [52] and Pugliese et al. [53], this study revealed that veterinarians with prior experience in performing euthanasia showed higher levels of agreement with euthanasia decisions in the case where the clinical condition deteriorated despite prolonged treatment (case 11) and the case involving a hereditary disease that prevented feeding (case 3). This suggests that veterinarians with prior experience in euthanasia consider euthanasia to be an ethically justifiable option in cases with clear clinical justifications, such as ineffective treatment and impaired quality of life. Additionally, the veterinarians viewed euthanasia as a humane method to end suffering. The fact that approximately 31% of the participants had never previously performed euthanasia on pet animals (Table 1) suggests that some of the assessments may have been hypothetical. However, the inclusion of participants without experience in performing euthanasia allowed for the evaluation of opinions regarding the scenarios among veterinarians with varying levels of experience.

In this study, 66 veterinarians reported that euthanasia should generally not be performed (Table 1), and their responses were included in the analyses of the individual clinical scenarios. Their general views on euthanasia may have influenced their levels of agreement with the decisions presented in these scenarios. However, the extent of this potential influence was not specifically examined. Future studies could investigate whether general views on euthanasia are associated with agreement levels across different clinical scenarios.

The analysis examining the relationship between years of clinical experience and previous euthanasia experience revealed no statistically significant association between these variables (χ^2^(3) = 1.068, *p* = 0.785). Further research is needed to explore the factors that may influence veterinarians’ exposure to euthanasia in clinical practice.

## 5. Conclusions

This is the first study to evaluate veterinarians’ levels of agreement with euthanasia decisions for companion animals among clinical veterinarians in Türkiye in various clinical scenarios, as well as the factors associated with these decisions. The findings of this study indicated that euthanasia decisions are correlated with the animal species and case characteristics and the individual and professional characteristics and ethical attitudes of veterinarians. In this study, veterinarians considered euthanasia as a necessary but difficult procedure but did not adopt it as a routine practice. These findings indicate that veterinarians’ assessments of euthanasia may vary according to animal species and case characteristics. However, the underlying reasons for these differences were not directly investigated in this study.

Veterinarians’ assessments of euthanasia did not differ significantly according to gender or workplace. However, the absence of statistically significant differences does not indicate that veterinarians base their decisions exclusively on professional criteria. In contrast, significant differences in assessments of euthanasia were observed according to pet ownership, professional experience, and previous experience with euthanasia. These findings suggest that veterinarians’ assessments of euthanasia may be associated with individual experiences as well as clinical and professional characteristics.

Thus, the decision on euthanasia is a multidimensional process involving the simultaneous consideration of clinical, ethical, and individual factors. Future qualitative studies must focus on the various case scenarios addressed in this research by performing detailed examinations and elucidating the ethical, professional, and individual rationales that shape euthanasia decisions, which can prove beneficial for pets, veterinarians, and animal owners.

This study has several limitations. Although the survey remained open for 10 months, 374 veterinarians participated. Recruitment through social media and professional networks using voluntary participation may have introduced selection bias and limited the representativeness of the sample. Furthermore, the lack of comparable national data on the demographic and professional characteristics of veterinarians in Türkiye prevented us from determining whether the sample reflects the wider veterinary population. In addition, the study assessed veterinarians’ agreement with euthanasia in hypothetical scenarios rather than their actual clinical decision-making. These limitations should be considered when interpreting the findings.

## Figures and Tables

**Table 1:** Sociodemographic data of the participants and their attitudes toward euthanasia.

Variable		*n*	%
Gender			
	Female	162	43.3
	Male	212	56.7
Age			
	22–33	169	45.2
	34–44	149	39.8
	45–55	43	11.5
	≥56	13	3.5
Work environment			
	Veterinary practice	273	73.0
	Polyclinic	48	12.8
	Animal hospital	53	14.2
Clinical experience (years)			
	<5	93	24.9
	5–10	144	38.5
	11–21	106	28.3
	≥22	31	8.3
Pet ownership			
	No	110	29.4
	Yes	264	70.6
Approach to euthanasia in pets			
A routine procedure		-	-
A procedure necessary for animal welfare		44	11.8
A necessary but difficult-to-perform procedure		202	54.0
A procedure that causes distress and doubt		62	16.6
A procedure that a veterinarian should not perform		66	17.6
The situation of performing euthanasia on a pet			
	No	116	31.0
	Yes	258	69.0
The situation of euthanizing one’s own pet			
	No	170	85.4
	Yes	29	14.6

**Table 2:** Comparison of agreement with euthanasia decision requests by veterinarians according to animal species.

Case	Cat, Median (Q1–Q3)	Dog, Median (Q1–Q3)	Small Mammal, Median (Q1–Q3)	Friedman χ^2^	*p*	Significant Pairwise Comparisons
Case 1	2.50 (1.00–3.00)	3.00 (2.00–4.00)	3.00 (1.00–4.00)	202.17	**<0.001**	Cat–Dog; Dog–Small mammal
Case 2	2.00 (1.00–2.00)	3.00 (2.00–3.00)	3.00 (1.00–3.00)	80.77	**<0.001**	Cat–Dog; Dog–Small mammal
Case 3	4.00 (3.00–4.00)	4.00 (3.00–4.00)	4.00 (3.00–4.00)	2.00	0.368	**-**
Case 4	1.00 (1.00–2.00)	1.00 (1.00–2.00)	2.00 (1.00–2.00)	118.22	**<0.001**	Cat–Small mammal; Dog–Small mammal
Case 5	2.00 (1.00–3.00)	2.00 (1.00–3.00)	2.00 (1.00–3.00)	58.72	**<0.001**	Cat–Small mammal; Dog–Small mammal
Case 6	2.00 (1.00–3.00)	2.00 (1.00–3.00)	2.00 (1.00–3.00)	82.90	**<0.001**	Cat–Small mammal; Dog–Small mammal
Case 7	2.00 (1.00–3.00)	2.00 (1.00–3.00)	2.00 (1.00–3.00)	24.35	**<0.001**	Cat–Dog; Cat–Small mammal
Case 8	2.00 (1.00–3.00)	3.00 (1.00–4.00)	2.00 (1.00–3.00)	215.28	**<0.001**	Cat–Dog; Dog–Small mammal
Case 9	3.00 (2.00–4.00)	3.00 (2.00–4.00)	3.00 (2.00–4.00)	9.56	**0.008**	Cat–Small mammal; Dog–Small mammal
Case 10	3.00 (2.00–4.00)	3.00 (2.00–4.00)	3.00 (2.00–4.00)	0.50	0.779	**-**
Case 11	4.00 (3.00–4.00)	4.00 (3.00–4.00)	4.00 (3.00–4.00)	3.50	0.174	**-**
Case 12	2.00 (1.00–2.00)	2.00 (1.00–2.00)	2.00 (1.00–3.00)	92.12	**<0.001**	Cat–Small mammal; Dog–Small mammal
Case 13	2.00 (1.00–2.00)	2.00 (1.00–2.00)	2.00 (1.00–3.00)	126.09	**<0.001**	Cat–Small mammal; Dog–Small mammal
Case 14	4.00 (3.00–4.25)	4.00 (3.00–4.25)	4.00 (3.00–5.00)	3.60	0.165	**-**

Values are presented as median (Q1–Q3), where Q1 and Q3 represent the 25th and 75th percentiles, respectively. Differences among animal species were assessed using the Friedman test. Following statistically significant Friedman tests, pairwise comparisons were performed using the Durbin–Conover procedure. A *p*-value < 0.05 was considered statistically significant. Statistically significant differences are indicated in bold.

**Table 3:** Levels of agreement with euthanasia decisions according to pet ownership status.

Case	Species	No Pet Ownership Median (Q1–Q3)	Pet Ownership Median (Q1–Q3)	U	*p*
1	Cat	3.00 (2.00–4.00)	2.00 (1.00–3.00)	10,624.50	**<0.001**
	Dog	4.00 (3.00–4.00)	3.00 (2.00–4.00)	10,241.00	**<0.001**
	Small mammal	3.00 (2.00–4.00)	2.00 (1.00–3.00)	11,170.00	**<0.001**
2	Cat	2.00 (2.00–3.00)	2.00 (1.00–2.00)	10,483.00	**<0.001**
	Dog	3.00 (2.00–4.00)	2.00 (1.00–3.00)	9630.00	**<0.001**
	Small mammal	2.00 (2.00–3.00)	2.00 (1.00–2.00)	10,401.50	**<0.001**
3	Cat	4.00 (3.00–4.00)	3.00 (3.00–4.00)	11,972.00	**0.004**
	Dog	4.00 (3.00–4.00)	3.00 (3.00–4.00)	11,909.50	**0.003**
	Small mammal	4.00 (3.00–4.00)	3.00 (3.00–4.00)	11,909.50	**0.003**
4	Cat	2.00 (1.00–2.00)	1.00 (1.00–2.00)	9978.00	**<0.001**
	Dog	2.00 (1.00–2.00)	1.00 (1.00–2.00)	9856.00	**<0.001**
	Small mammal	2.00 (1.00–3.25)	1.00 (1.00–2.00)	9467.00	**<0.001**
5	Cat	3.00 (2.00–3.00)	2.00 (1.00–3.00)	11,479.00	**<0.001**
	Dog	3.00 (2.00–3.00)	2.00 (1.00–3.00)	11,514.50	**<0.001**
	Small mammal	3.00 (2.00–4.00)	2.00 (1.00–3.00)	11,103.50	**<0.001**
6	Cat	2.00 (2.00–3.00)	2.00 (1.00–3.00)	10,244.50	**<0.001**
	Dog	2.00 (2.00–3.00)	2.00 (1.00–3.00)	10,222.00	**<0.001**
	Small mammal	3.00 (2.00–4.00)	2.00 (1.00–3.00)	9678.00	**<0.001**
7	Cat	2.00 (2.00–3.00)	2.00 (1.00–3.00)	10,789.00	**<0.001**
	Dog	3.00 (2.00–4.00)	2.00 (1.00–3.00)	9960.50	**<0.001**
	Small mammal	3.00 (2.00–4.00)	2.00 (1.00–3.00)	10,784.50	**<0.001**
8	Cat	2.00 (2.00–3.00)	2.00 (1.00–3.00)	10,542.00	**<0.001**
	Dog	4.00 (2.00–4.00)	2.00 (1.00–4.00)	9653.00	**<0.001**
	Small mammal	3.00 (2.00–3.00)	2.00 (1.00–3.00)	10,612.50	**<0.001**
9	Cat	4.00 (2.00–4.00)	3.00 (1.00–4.00)	10,180.00	**<0.001**
	Dog	4.00 (2.00–4.00)	3.00 (1.00–4.00)	10,165.00	**<0.001**
	Small mammal	4.00 (2.00–4.00)	3.00 (1.00–4.00)	10,350.50	**<0.001**
10	Cat	4.00 (2.75–4.00)	3.00 (1.00–4.00)	11,321.00	**<0.001**
	Dog	4.00 (2.75–4.00)	3.00 (1.00–4.00)	11,234.50	**<0.001**
	Small mammal	4.00 (2.75–4.00)	3.00 (1.00–4.00)	11,376.00	**<0.001**
11	Cat	4.00 (4.00–4.00)	4.00 (3.00–4.00)	14,011.00	0.546
	Dog	4.00 (4.00–4.00)	4.00 (3.00–4.00)	13,820.00	0.407
	Small mammal	4.00 (4.00–4.00)	4.00 (3.00–4.00)	14,112.50	0.629
12	Cat	2.00 (2.00–2.00)	1.00 (1.00–2.00)	10,049.00	**<0.001**
	Dog	2.00 (2.00–2.00)	1.00 (1.00–2.00)	10,014.50	**<0.001**
	Small mammal	2.00 (2.00–3.00)	1.00 (1.00–2.00)	10,070.50	**<0.001**
13	Cat	2.00 (2.00–3.00)	1.00 (1.00–2.00)	9962.00	**<0.001**
	Dog	2.00 (2.00–3.00)	1.00 (1.00–2.00)	9944.00	**<0.001**
	Small mammal	2.50 (2.50–4.00)	1.00 (1.00–3.00)	9506.50	**<0.001**
14	Cat	4.00 (4.00–5.00)	4.00 (3.00–4.00)	12,658.00	**0.036**
	Dog	4.00 (4.00–5.00)	4.00 (3.00–4.00)	12,614.50	**0.032**
	Small mammal	4.00 (4.00–5.00)	4.00 (3.00–4.00)	12,490.00	**0.022**

Values are presented as the median (Q1–Q3), where Q1 and Q3 represent the 25th and 75th percentiles, respectively. Differences between veterinarians with and without pet ownership were assessed using the Mann–Whitney U test. A *p*-value < 0.05 was considered statistically significant. Statistically significant differences are indicated in bold.

**Table 4:** Agreement with euthanasia decisions according to years of professional experience.

Case	Species	<5 Median (Q1–Q3)	5–10 Median (Q1–Q3)	11–21 Median (Q1–Q3)	≥22 Median (Q1–Q3)	χ^2^	df	*p*
1	Cat	2.00 (1.00–3.00) ^a^	3.00 (2.00–4.00) ^b^	3.00 (2.00–4.00) ^b^	2.00 (2.00–3.00) ^a^	27.42	3	**<0.001**
	Dog	3.00 (1.00–4.00) ^a^	3.00 (2.00–4.00) ^ab^	4.00 (2.00–4.00) ^b^	2.00 (2.00–3.00) ^a^	20.39	3	**<0.001**
	Small mammal	2.00 (1.00–3.00) ^a^	3.00 (2.00–4.00) ^b^	3.00 (2.00–4.00) ^b^	2.00 (2.00–3.00) ^a^	22.00	3	**<0.001**
2	Cat	1.00 (1.00–2.00) ^a^	2.00 (1.25–3.00) ^b^	2.00 (2.00–3.00) ^b^	1.00 (1.00–2.00) ^a^	27.43	3	**<0.001**
	Dog	1.00 (1.00–2.00) ^a^	2.00 (1.25–3.00) ^b^	2.00 (2.00–3.00) ^b^	1.00 (1.00–2.00) ^a^	30.77	3	**<0.001**
	Small mammal	1.00 (1.00–2.00) ^a^	2.00 (1.00–3.00) ^b^	2.00 (2.00–3.00) ^b^	1.00 (1.00–2.00) ^a^	27.74	3	**<0.001**
3	Cat	3.00 (2.00–4.00) ^ab^	4.00 (3.00–4.00) ^ab^	4.00 (4.00–4.00) ^a^	3.00 (3.00–4.00) ^b^	7.93	3	**0.048**
	Dog	3.00 (2.00–4.00)	4.00 (3.00–4.00)	4.00 (4.00–4.00)	3.00 (3.00–4.00)	7.72	3	0.052
	Small mammal	3.00 (2.00–4.00)	4.00 (3.00–4.00)	4.00 (4.00–4.00)	3.00 (3.00–4.00)	7.72	3	0.052
4	Cat	1.00 (1.00–2.00) ^a^	2.00 (1.00–2.00) ^b^	2.00 (2.00–2.00) ^b^	1.00 (1.00–2.00) ^a^	21.54	3	**<0.001**
	Dog	1.00 (1.00–2.00) ^a^	2.00 (1.00–2.00) ^b^	2.00 (2.00–2.00) ^b^	1.00 (1.00–2.00) ^a^	20.40	3	**<0.001**
	Small mammal	1.00 (1.00–2.00) ^a^	2.00 (1.00–2.00) ^b^	2.00 (2.00–3.00) ^b^	1.00 (1.00–2.00) ^a^	16.73	3	**<0.001**
5	Cat	1.00 (1.00–3.00) ^a^	2.00 (1.25–3.00) ^b^	3.00 (3.00–3.00) ^b^	1.00 (1.00–3.00)	18.80	3	**<0.001**
	Dog	1.00 (1.00–3.00) ^a^	2.00 (1.25–3.00) ^b^	3.00 (3.00–3.00) ^b^	1.00 (1.00–3.00)	19.03	3	**<0.001**
	Small mammal	2.00 (1.00–3.00) ^a^	2.00 (2.00–3.00) ^b^	3.00 (3.00–3.00) ^b^	1.00 (1.00–3.00)	17.33	3	**<0.001**
6	Cat	2.00 (1.00–3.00) ^ab^	2.00 (1.00–3.00) ^a^	2.00 (2.00–3.00) ^a^	1.00 (1.00–2.00) ^b^	15.40	3	**0.002**
	Dog	2.00 (1.00–3.00) ^ab^	2.00 (1.00–3.00) ^a^	2.00 (2.00–3.00) ^a^	1.00 (1.00–2.00) ^b^	15.69	3	**0.001**
	Small mammal	2.00 (1.00–3.00) ^ab^	2.00 (1.00–3.00) ^bc^	2.00 (2.00–4.00) ^c^	1.00 (1.00–2.00) ^a^	18.15	3	**<0.001**
7	Cat	2.00 (1.00–3.00) ^ab^	2.00 (2.00–3.00) ^bc^	2.00 (2.00–3.00) ^c^	1.00 (1.00–2.00) ^a^	23.23	3	**<0.001**
	Dog	2.00 (1.00–3.00) ^ab^	2.00 (2.00–3.00) ^bc^	3.00 (3.00–3.00) ^c^	1.00 (1.00–2.00) ^a^	24.51	3	**<0.001**
	Small mammal	2.00 (1.00–3.00) ^ab^	2.00 (2.00–3.00) ^a^	2.00 (2.00–3.00) ^a^	1.00 (1.00–2.00) ^b^	20.37	3	**<0.001**
8	Cat	1.00 (1.00–2.00) ^a^	2.00 (1.00–3.00) ^b^	2.00 (2.00–3.00) ^b^	1.00 (1.00–3.00) ^ab^	30.64	3	**<0.001**
	Dog	1.00 (1.00–3.00) ^a^	3.00 (1.00–4.00) ^b^	3.50 (3.50–4.00) ^b^	1.00 (1.00–3.00) ^a^	40.30	3	**<0.001**
	Small mammal	1.00 (1.00–3.00) ^a^	2.00 (1.00–3.00) ^b^	2.00 (2.00–3.00) ^c^	1.00 (1.00–3.00) ^ab^	30.18	3	**<0.001**
9	Cat	3.00 (1.00–3.50)	3.00 (1.00–4.00)	3.00 (3.00–4.00)	2.00 (2.00–3.00)	7.52	3	0.057
	Dog	3.00 (1.00–3.50)	3.00 (1.00–4.00)	3.00 (3.00–4.00)	2.00 (2.00–3.00)	7.43	3	0.059
	Small mammal	3.00 (1.00–4.00) ^ab^	3.00 (1.00–4.00) ^ab^	3.00 (3.00–4.00) ^a^	2.00 (2.00–3.00) ^b^	8.48	3	**0.037**
10	Cat	3.00 (1.00–4.00) ^a^	3.50 (2.00–4.00) ^ab^	4.00 (4.00–4.00) ^b^	3.00 (3.00–4.00) ^a^	17.90	3	**<0.001**
	Dog	3.00 (1.00–4.00) ^a^	3.50 (2.00–4.00) ^ab^	4.00 (4.00–4.00) ^b^	3.00 (3.00–4.00) ^a^	17.21	3	**<0.001**
	Small mammal	3.00 (1.00–4.00) ^a^	4.00 (2.00–4.00) ^ab^	4.00 (4.00–4.00) ^b^	3.00 (3.00–4.00) ^a^	17.35	3	**<0.001**
11	Cat	4.00 (3.00–4.00)	4.00 (2.00–4.00)	4.00 (4.00–4.00)	4.00 (4.00–4.00)	4.65	3	0.200
	Dog	4.00 (3.00–4.00)	4.00 (2.00–4.00)	4.00 (4.00–4.00)	4.00 (4.00–4.00)	4.83	3	0.185
	Small mammal	4.00 (3.00–4.00)	4.00 (2.00–4.00)	4.00 (4.00–4.00)	4.00 (4.00–4.00)	4.91	3	0.179
12	Cat	1.00 (1.00–2.00) ^a^	2.00 (1.00–2.00) ^b^	2.00 (2.00–2.00) ^b^	1.00 (1.00–2.00) ^a^	25.06	3	**<0.001**
	Dog	1.00 (1.00–2.00) ^a^	2.00 (1.00–2.00) ^b^	2.00 (2.00–2.00) ^b^	1.00 (1.00–2.00) ^a^	25.79	3	**<0.001**
	Small mammal	1.00 (1.00–2.00) ^a^	2.00 (1.00–3.00) ^b^	2.00 (2.00–3.00) ^b^	1.00 (1.00–2.00) ^a^	23.94	3	**<0.001**
13	Cat	1.00 (1.00–2.00) ^a^	2.00 (1.00–2.00) ^b^	2.00 (2.00–2.00) ^b^	1.00 (1.00–2.00) ^a^	25.43	3	**<0.001**
	Dog	1.00 (1.00–2.00) ^a^	2.00 (1.00–2.00) ^b^	2.00 (2.00–2.00) ^b^	1.00 (1.00–2.00) ^a^	25.20	3	**<0.001**
	Small mammal	1.00 (1.00–3.00) ^a^	2.00 (1.00–3.00) ^b^	2.00 (2.00–4.00) ^b^	1.00 (1.00–2.00) ^a^	21.72	3	**<0.001**
14	Cat	4.00 (3.00–5.00)	4.00 (4.00–4.00)	4.00 (4.00–4.25)	4.00 (4.00–4.00)	5.16	3	0.160
	Dog	4.00 (3.00–5.00)	4.00 (4.00–4.00)	4.00 (4.00–4.25)	4.00 (4.00–4.00)	5.02	3	0.170
	Small mammal	4.00 (3.00–5.00)	4.00 (4.00–4.00)	4.00 (4.00–4.25)	4.00 (4.00–4.00)	4.48	3	0.214

Values are presented as the median (Q1–Q3), where Q1 and Q3 represent the 25th and 75th percentiles, respectively. Differences among professional experience groups were assessed using the Kruskal–Wallis test. Different superscript letters (a, b, c) indicate statistically significant differences between groups. A *p*-value < 0.05 was considered statistically significant. Statistically significant differences are indicated in bold.

**Table 5:** Agreement with euthanasia decisions based on prior experience with performing euthanasia.

Case	Species	No Previous Euthanasia Experience Median (Q1–Q3)	Previous Euthanasia Experience Median (Q1–Q3)	U	*p*
3	Cat	3.00 (3.00–4.00)	4.00 (3.00–4.00)	12,942.00	**0.025**
	Dog	3.00 (3.00–4.00)	4.00 (3.00–4.00)	12,917.00	**0.023**
	Small mammal	3.00 (3.00–4.00)	4.00 (3.00–4.00)	12,917.00	**0.023**
8	Cat	2.00 (2.00–3.00)	2.00 (1.00–3.00)	12,210.00	**0.003**
	Small mammal	2.00 (2.00–3.00)	2.00 (1.00–3.00)	12,485.50	**0.007**
11	Cat	4.00 (3.00–4.00)	4.00 (4.00–4.00)	10,173.00	**<0.001**
	Dog	4.00 (3.00–4.00)	4.00 (4.00–4.00)	10,095.00	**<0.001**
	Small mammal	4.00 (3.00–4.00)	4.00 (4.00–4.00)	10,268.00	**<0.001**
14	Cat	4.00 (4.00–5.00)	4.00 (3.00–4.00)	13,146.50	**0.043**
	Dog	4.00 (4.00–5.00)	4.00 (3.00–4.00)	13,099.00	**0.038**
	Small mammal	4.00 (4.00–5.00)	4.00 (3.00–4.00)	13,173.00	**0.046**

Values are presented as the median (Q1–Q3), where Q1 and Q3 represent the 25th and 75th percentiles, respectively. Differences between veterinarians with and without previous euthanasia experience were assessed using the Mann–Whitney U test. A *p*-value < 0.05 was considered statistically significant. Statistically significant differences are indicated in bold.

## Data Availability

The data supporting the findings of this study are not publicly available due to privacy and ethical restrictions but may be available from the corresponding author upon reasonable request, subject to appropriate ethical approval and data protection requirements.

## Supplementary Materials

The following supporting information can be downloaded at https://www.mdpi.com/article/10.3390/ani16213349/s1. Supplementary Table S1. Agreement with euthanasia decisions according to gender. Supplementary Table S2. Agreement with euthanasia decision requests by veterinarians according to their work environments.

## Abbreviation

The following abbreviation is used in this manuscript:

MAF	Ministry of Agriculture and Forestry
