# Supplementary material for: Clinical Veterinarian Perspectives on Euthanasia for Pets
Source: Animals (Basel). 2026 Oct 23;16(21):3349. doi: 10.3390/ani16213349 (PMC13762262; doi:10.3390/ani16213349)
Supplement: Supplementary file 1 [file animals-16-03349-s001.zip › animals-4614686-supplementary.pdf]

Supplementary Table S1. Agreement with euthanasia decisions according to gender.

Case	Species	Female, Median (Q1–Q3)	Male, Median (Q1–Q3)	U	p
1	Cat	3.00 (2.00–4.00)	2.00 (1.00–3.00)	16118.50	0.294
	Dog	3.00 (2.00–4.00)	3.00 (2.00–4.00)	16596.00	0.566
	Small mammal	3.00 (2.00–4.00)	3.00 (1.00–3.00)	16449.00	0.472
2	Cat	2.00 (1.00–3.00)	2.00 (1.00–2.00)	16060.00	0.254
	Dog	2.00 (1.00–3.00)	2.00 (1.00–3.00)	16198.50	0.326
	Small mammal	2.00 (1.00–3.00)	2.00 (1.00–2.00)	15995.00	0.228
3	Cat	4.00 (3.00–4.00)	4.00 (3.00–4.00)	16278.00	0.354
	Dog	4.00 (3.00–4.00)	4.00 (3.00–4.00)	16235.50	0.332
	Small mammal	4.00 (3.00–4.00)	4.00 (3.00–4.00)	16235.50	0.332
4	Cat	2.00 (1.00–2.00)	1.00 (1.00–2.00)	15789.00	0.137
	Dog	2.00 (1.00–2.00)	1.00 (1.00–2.00)	15849.00	0.155
	Small mammal	2.00 (1.00–2.00)	1.00 (1.00–2.00)	16211.50	0.315
5	Cat	2.00 (1.00–3.00)	2.00 (1.00–3.00)	16569.00	0.543
	Dog	2.00 (1.00–3.00)	2.00 (1.00–3.00)	16618.00	0.577
	Small mammal	2.00 (1.00–3.00)	2.00 (1.00–3.00)	17140.00	0.975
6	Cat	2.00 (1.00–3.00)	2.00 (1.00–3.00)	16206.00	0.329
	Dog	2.00 (1.00–3.00)	2.00 (1.00–3.00)	16000.00	0.236
	Small mammal	2.00 (1.00–3.00)	2.00 (1.00–3.00)	16427.00	0.456
7	Cat	2.00 (1.00–3.00)	2.00 (1.00–3.00)	16589.50	0.559
	Dog	2.00 (1.00–3.00)	2.00 (1.00–3.00)	16151.50	0.308
	Small mammal	2.00 (1.75–3.00)	2.00 (1.00–3.00)	16601.00	0.568
8	Cat	2.00 (1.00–3.00)	2.00 (1.00–3.00)	15810.00	0.167
	Dog	3.00 (1.00–4.00)	2.00 (1.00–4.00)	15638.00	0.121
	Small mammal	2.00 (1.00–3.00)	2.00 (1.00–3.00)	16159.50	0.306
9	Cat	3.00 (2.00–4.00)	3.00 (2.00–4.00)	16647.00	0.601
	Dog	3.00 (2.00–4.00)	3.00 (2.00–4.00)	16614.00	0.578
	Small mammal	3.00 (2.00–4.00)	3.00 (2.00–4.00)	16692.50	0.632
10	Cat	3.00 (2.00–4.00)	3.00 (2.00–4.00)	16870.50	0.760
	Dog	3.00 (2.00–4.00)	3.00 (2.00–4.00)	16892.00	0.777
	Small mammal	3.00 (2.00–4.00)	3.00 (2.00–4.00)	16832.50	0.731
11	Cat	4.00 (3.00–4.00)	4.00 (3.00–4.00)	16879.50	0.750
	Dog	4.00 (3.00–4.00)	4.00 (3.00–4.00)	16757.00	0.651
	Small mammal	4.00 (3.00–4.00)	4.00 (3.00–4.00)	16874.00	0.746
12	Cat	2.00 (1.00–2.00)	2.00 (1.00–2.00)	16850.00	0.737
	Dog	2.00 (1.00–2.00)	2.00 (1.00–2.00)	16807.50	0.703
	Small mammal	2.00 (1.00–3.00)	2.00 (1.00–3.00)	17058.50	0.908
13	Cat	2.00 (1.00–2.00)	2.00 (1.00–2.00)	16006.50	0.225
	Dog	2.00 (1.00–2.00)	2.00 (1.00–2.00)	16021.50	0.231
	Small mammal	2.00 (1.00–3.00)	2.00 (1.00–3.00)	16287.00	0.368
14	Cat	4.00 (3.75–5.00)	4.00 (3.00–4.00)	16694.50	0.620
	Dog	4.00 (3.75–5.00)	4.00 (3.00–4.00)	16623.50	0.570
	Small mammal	4.00 (3.75–5.00)	4.00 (3.00–4.00)	16806.00	0.704

Values are presented as median (Q1–Q3), where Q1 and Q3 represent the 25th and 75th percentiles, respectively. Differences between female and male veterinarians were assessed using the Mann–Whitney U test. A p-value < 0.05 was considered statistically significant.

Supplementary Table S2. Agreement with euthanasia decisions requests by veterinarians according to their work environments.

Case	Species	Veterinary practice Median (Q1–Q3)	Polyclinic Median (Q1–Q3)	Animal hospital Median (Q1–Q3)	χ^2	df	p
1	Cat	2.00 (1.00–4.00)	2.00 (1.25–3.00)	3.00 (2.00–3.00)	0.68	2	0.711
	Dog	3.00 (2.00–4.00)	3.00 (2.00–4.00)	3.00 (2.00–4.00)	2.49	2	0.288
	Small mammal	3.00 (1.00–4.00)	3.00 (2.00–3.00)	3.00 (1.50–3.00)	0.05	2	0.976
2	Cat	2.00 (1.00–3.00)	2.00 (1.00–2.75)	2.00 (1.00–2.00)	0.14	2	0.933
	Dog	2.00 (1.00–3.00)	2.00 (1.00–3.00)	2.00 (1.00–2.00)	0.92	2	0.632
	Small mammal	2.00 (1.00–3.00)	2.00 (1.00–2.75)	2.00 (1.00–2.00)	0.20	2	0.903
3	Cat	4.00 (3.00–4.00)	3.50 (3.00–4.00)	3.00 (3.00–4.00)	0.01	2	0.995
	Dog	4.00 (3.00–4.00)	3.50 (3.00–4.00)	3.00 (3.00–4.00)	0.01	2	0.994
	Small mammal	4.00 (3.00–4.00)	3.50 (3.00–4.00)	3.00 (3.00–4.00)	0.01	2	0.994
4	Cat	1.00 (1.00–2.00)	1.00 (1.00–2.00)	2.00 (1.00–2.00)	0.47	2	0.790
	Dog	1.00 (1.00–2.00)	1.00 (1.00–2.00)	2.00 (1.00–2.00)	0.44	2	0.802
	Small mammal	1.00 (1.00–2.00)	1.00 (1.00–2.00)	2.00 (1.00–2.00)	0.33	2	0.847
5	Cat	2.00 (1.00–3.00)	2.00 (1.00–3.00)	2.00 (1.00–3.00)	0.17	2	0.916
	Dog	2.00 (1.00–3.00)	2.00 (1.00–3.00)	2.00 (1.00–3.00)	0.17	2	0.918
	Small mammal	2.00 (1.00–3.00)	3.00 (1.00–3.00)	2.00 (1.50–3.00)	0.79	2	0.675
6	Cat	2.00 (1.00–3.00)	2.00 (1.00–2.00)	2.00 (1.00–3.00)	0.31	2	0.858
	Dog	2.00 (1.00–3.00)	2.00 (1.00–2.00)	2.00 (1.00–3.00)	0.31	2	0.856
	Small mammal	2.00 (1.00–3.00)	2.00 (1.00–3.00)	2.00 (1.50–3.00)	0.09	2	0.957
7	Cat	2.00 (1.00–3.00)	2.00 (1.00–3.00)	2.00 (2.00–3.00)	2.34	2	0.310
	Dog	2.00 (1.00–3.00)	2.00 (1.00–3.00)	2.00 (1.50–3.00)	2.46	2	0.292
	Small mammal	2.00 (1.00–3.00)	2.00 (1.00–3.00)	2.00 (2.00–3.00)	1.99	2	0.370
8	Cat	2.00 (1.00–3.00)	2.00 (1.00–3.00)	2.00 (1.00–3.00)	0.87	2	0.646
	Dog	2.00 (1.00–4.00)	3.00 (1.00–4.00)	3.00 (1.00–3.00)	1.15	2	0.563
	Small mammal	2.00 (1.00–3.00)	2.00 (1.00–3.00)	2.00 (1.00–3.00)	0.95	2	0.622
9	Cat	3.00 (2.00–4.00)	3.00 (1.00–4.00)	3.00 (2.00–3.50)	0.42	2	0.809
	Dog	3.00 (2.00–4.00)	3.00 (1.00–4.00)	3.00 (2.00–3.50)	0.39	2	0.823
	Small mammal	3.00 (2.00–4.00)	3.00 (1.00–4.00)	3.00 (2.00–3.50)	0.39	2	0.823
10	Cat	3.00 (2.00–4.00)	4.00 (2.00–4.00)	3.00 (2.00–4.00)	1.10	2	0.577
	Dog	3.00 (2.00–4.00)	4.00 (2.00–4.00)	3.00 (2.00–4.00)	1.07	2	0.585
	Small mammal	3.00 (2.00–4.00)	4.00 (2.00–4.00)	3.00 (2.00–4.00)	1.03	2	0.597
11	Cat	4.00 (3.00–4.00)	4.00 (4.00–4.00)	4.00 (3.00–4.00)	2.66	2	0.264
	Dog	4.00 (3.00–4.00)	4.00 (4.00–4.00)	4.00 (3.00–4.00)	2.54	2	0.281
	Small mammal	4.00 (3.00–4.00)	4.00 (4.00–4.00)	4.00 (3.00–4.00)	2.54	2	0.281
12	Cat	2.00 (1.00–2.00)	2.00 (1.00–2.00)	2.00 (1.00–2.00)	0.14	2	0.934
	Dog	2.00 (1.00–2.00)	2.00 (1.00–2.00)	2.00 (1.00–2.00)	0.15	2	0.926
	Small mammal	2.00 (1.00–3.00)	2.00 (1.00–3.00)	2.00 (1.00–3.00)	0.04	2	0.983
13	Cat	2.00 (1.00–2.00)	1.50 (1.00–2.00)	2.00 (1.00–2.00)	2.15	2	0.342
	Dog	2.00 (1.00–2.00)	1.50 (1.00–2.00)	2.00 (1.00–2.00)	2.15	2	0.341
	Small mammal	2.00 (1.00–3.00)	2.00 (1.00–3.00)	2.00 (1.00–3.00)	0.56	2	0.755
14	Cat	4.00 (3.00–4.00)	4.00 (4.00–5.00)	4.00 (3.00–5.00)	2.43	2	0.297
	Dog	4.00 (3.00–4.00)	4.00 (4.00–5.00)	4.00 (3.00–5.00)	2.51	2	0.285
	Small mammal	4.00 (3.00–4.00)	4.00 (4.00–5.00)	4.00 (3.00–5.00)	2.20	2	0.334

Values are presented as median (Q1–Q3), where Q1 and Q3 represent the 25th and 75th percentiles, respectively. Differences among work environments were assessed using the Kruskal–Wallis test. A p-value < 0.05 was considered statistically significant.
